# A Real-World Clinical Evaluation of Ganciclovir Exposure and Cytomegalovirus Viremia Outcomes After Adult Kidney Transplantation

**DOI:** 10.1097/FTD.0000000000001363

**Published:** 2025-07-21

**Authors:** Lukas K. van Vugt, Rawan Saleh, Dennis A. Hesselink, Brenda C. M. de Winter

**Affiliations:** * Erasmus MC Transplant Institute;; † Division of Nephrology and Transplantation, Department of Internal Medicine, Erasmus MC, University Medical Centre Rotterdam; and; ‡ Department of Hospital Pharmacy, Erasmus MC, University Medical Centre Rotterdam, Rotterdam, the Netherlands.

**Keywords:** valganciclovir, therapeutic drug monitoring, cytomegalovirus, kidney transplantation

## Abstract

**Background:**

The efficacy and toxicity of valganciclovir prophylaxis are determined by ganciclovir (GCV) exposure. As GCV is predominantly eliminated through glomerular filtration, kidney transplant recipients are at risk of both under- and overexposure. Kidney function is dynamic and GCV exposure may be off-target shortly after kidney transplantation and in kidney transplant recipients with delayed graft function (DGF). However, the extent of GCV under- and overexposure in the early phase after kidney transplantation has not been well described.

**Methods:**

This was a retrospective, single-center analysis of adult patients who received valganciclovir prophylaxis and underwent at least one predose steady-state GCV concentration measurement during the first month after kidney transplantation. Data on GCV measurements, target attainment, and clinical outcomes, including cytomegalovirus (CMV) viremia and leukopenia, were collected and analyzed.

**Results:**

Overall, 353 patients were included, with 357 predose and steady-state GCV concentration measurements. GCV exposure was highly variable early after kidney transplantation, especially in patients with poorly functioning grafts and those undergoing dialysis. Overall, there was a large proportion of patients with GCV exposure below the target, especially those with higher estimated kidney function, despite many receiving high GCV dosages. However, despite the frequent occurrence of off-target GCV exposure, no association was observed between DGF, GCV exposure, and CMV viremia.

**Conclusions:**

In the early phase after kidney transplantation, exposure to GCV is highly variable among patients and underexposure to GCV occurs frequently. However, DGF was not associated with reduced exposure to GCV or CMV viremia, nor was early exposure to GCV correlated with CMV outcomes or leukopenia.

## Abstract 2

Supplemental Digital Content is Available in the Text.

## BACKGROUND

The introduction of valganciclovir (VGCV), a prodrug of ganciclovir (GCV), has reduced both the incidence and severity of cytomegalovirus (CMV)-related complications after kidney transplantation.^1^ However, breakthrough CMV infections may occur despite VGCV prophylaxis,^2^ and VGCV toxicity, most importantly leukopenia, frequently complicates its use.^3^ The incidence of breakthrough infections and leukopenia after VGCV is related to GCV under- and overexposure, respectively.^4^

GCV is predominantly eliminated through glomerular filtration.^5^ The glomerular filtration rate (GFR) should, therefore, be considered when dosing VGCV.^6^ Kidney transplant recipients are at risk of both under- and overexposure to GCV because of rapid restoration of kidney function after successful transplantation or transplant-related complications leading to a reduced GFR, respectively.^7^ However, pharmacokinetic studies investigating these estimated GFR (eGFR)-based prophylactic dose reductions are scarce, and large variability in GCV exposure has been described despite adjusting the VGCV dose for the eGFR.^4^ Moreover, the eGFR provides only an approximation of the actual GFR when serum creatinine production and clearance are in steady state, which is not the case early after kidney transplantation.^8,9^ In addition, GCV is cleared by hemodialysis, which may also cause variable exposure to GCV in case of delayed graft function (DGF).^7^ Adjusting the VGCV dose based on the eGFR may, therefore, not be appropriate shortly after transplantation and cause off-target GCV exposure.

DGF is most commonly defined as the necessity for dialysis in the first week after kidney transplantation, and it complicates approximately 30% of all kidney transplantations.^10^ DGF exhibits variable duration and a dynamic course.^11^ It is, therefore, likely that the occurrence of DGF complicates the dosing of VGCV. DGF has been associated with an increased risk of CMV-related complications.^12^ Moreover, eGFR-adjusted VGCV dose reductions during DGF have been suggested to be too high and cause reduced GCV exposure; however, the GCV exposure during DGF was not reported.^12^

GCV concentrations can be measured in the plasma, but therapeutic drug monitoring (TDM) is not routinely performed for this drug.^13^ At the Erasmus MC Rotterdam hospital, GCV concentrations are determined after kidney transplantation at the discretion of the treating nephrologist. Our clinical experience with GCV TDM is reported in the present study. The aim of this study was to assess GCV exposure early after kidney transplantation and to determine its association with DGF and the occurrence of CMV infection and GCV-related toxicity in a real-world setting.

## METHODS

### Study Design

This retrospective, single-center study was approved by the local medical ethical board of the Erasmus MC Rotterdam (METC-2021-0794) and classified as not subject to the Dutch Medical Research Involving Human Subjects Act.^14^ Informed consent of the individual patients was not required for this study because all patients had previously agreed to the use of their clinical data for research purposes. Clinical data were obtained from the hospital medical records and the Dutch National Organ Transplantation Registry (NOTR). All data were collected using an electronic web-based data-capture system. The collected data included recipient and donor characteristics, use of immunosuppressive medication and anti-CMV drugs, laboratory values, and medically important events (eg, patient survival, graft loss, DGF, and CMV viremia).

### Patients

Patients eligible for inclusion were identified through the hospital pharmacy laboratory as follows: a list of adult renal transplant patients with GCV measurements was obtained from the hospital pharmacy laboratory and crosschecked with their renal transplantation date to include only GCV concentrations that were measured in the first post-transplant month. The renal transplantation date was obtained from the NOTR. Patients were eligible for inclusion between January 2017 and December 2023 if they were 18 years of age or older, if they received VGCV prophylaxis for either CMV-positive donor (D+) and/or CMV-positive recipient (R+) transplantation and/or T-lymphocyte–depleting antibody therapy, and if they had at least one steady-state GCV concentration measured in the first month after transplantation. The included patients were followed up until the end of the first year after transplantation.

### Local Prophylactic Strategy

All recipients with either a CMV-seropositive status before transplantation (R+) or recipients of a CMV-seropositive donor kidney transplant (D+) received VGCV prophylaxis. In addition, patients who received T-lymphocyte–depleting antibody therapy received VGCV prophylaxis, regardless of the D+/R+ status. VGCV prophylaxis was initiated on the second day after transplantation. The standard prophylaxis duration was 3 months for seropositive recipients (both D-/R+ and D+/R+), 6 months for D+/R-recipients, and until the T-lymphocyte count exceeded 200 × 10^6^ cells/L for patients receiving T-lymphocyte–depleting therapy.

### Dosing Protocol

In patients with steady-state kidney function, the current eGFR-adjusted dosing advice for VGCV was followed.^15^ However, during the first week after kidney transplantation, when the use of eGFR is inappropriate because kidney function is not stable, the initial VGCV dose was 450 mg once daily. Based on serum creatinine dynamics and urine production after transplantation, this dose was continued or reduced to 450 mg every second day, 450 mg twice per week, or 450 mg once per week. Precise dose reduction was determined at the discretion of the treating nephrologist.

### Ganciclovir Measurements and Interpretation

No formal GCV TDM protocol was followed. Instead, plasma GCV concentration measurements were performed at the discretion of the treating nephrologist. The consulting hospital pharmacist interpreted the measurements as being obtained at a steady state and as a predose concentration below, within, or above the prophylactic range. The measurement was considered predose if it was obtained within 2 hours before the next VGCV dose. The GCV concentration was considered steady state when the VGCV dose remained unchanged for at least 3 consecutive days before the GCV concentration measurement. The lower limit of the prophylactic range was defined as 0.5 mg/L, which was derived from the in vitro IC50 of GCV in CMV.^16^ The upper limit of the prophylactic range was 5.0 mg/L and was based on in vitro toxicity.^17^ Depending on the measured concentration, the hospital pharmacist advised dose continuation or dose adjustment.

### Analytical Methods

Plasma CMV viral loads were assessed using a real-time CMV-DNA polymerase chain reaction (PCR) assay. Serum GCV concentrations were determined using a validated liquid chromatography assay coupled with mass spectrometry at a detection range of 0.1–10 mg/L.^18^

### Study Outcomes

The primary outcome of this study was GCV exposure early after kidney transplantation. For this purpose, both the median steady-state predose concentrations and the proportions of concentrations below, within, and above the target were related to the VGCV dose and compared between different eGFR ranges, which were based on eGFR-adjusted dosing ranges. In addition, the occurrence of CMV *primo* infection and reactivation was studied. Clinically relevant CMV replication was defined as producing at least one positive CMV PCR ≥50 IU/mL, which is the lower limit of detection. Patients were not routinely screened for CMV replication, but only in cases of clinical suspicion (eg, unexplained biochemical findings, viral symptoms, or organ-specific symptoms). If no CMV viral loads were obtained during follow-up, the patients were considered not to have experienced clinically relevant CMV replication. CMV replication was defined as asymptomatic viremia or symptomatic infection. Symptomatic infection was further classified as CMV syndrome (characterized by general symptoms of viral infections, including fever, malaise, and fatigue) and organ-specific CMV disease, for example, CMV colitis, retinitis, or hepatitis.^19^ CMV infection was also defined by its occurrence during prophylaxis or after cessation of prophylaxis. As a secondary outcome, the associations between DGF and GCV exposure, CMV viremia, and leukopenia were assessed. DGF was defined as the need for dialysis in the first week after kidney transplantation. A DGF episode lasted from the first day of dialysis to the spontaneous improvement in serum creatinine. Leukocyte counts were analyzed between the day of transplantation and 45 days after transplantation. Leukopenia was defined as a leukocyte count <3500 cells/μL, which is a commonly used threshold after adult kidney transplantation in observational studies.^20^ General outcomes after kidney transplantation, including patient survival and death-censored graft loss, were also reported.

### Statistical Analysis

Descriptive statistics were used to describe patient, donor, and immunosuppressive treatment characteristics. Continuous variables were presented as medians and interquartile ranges. Categorical variables are presented as proportions. The GCV concentrations were log-transformed. Log-transformed GCV concentrations were visually assessed for normality and compared between the 2 groups using an unpaired *t* test for normally distributed data and the Mann–Whitney *U* test for skewed data. The proportions between 2 or more groups were compared using Fisher exact test.

Multivariate logistic regression analysis was performed to evaluate the association between DGF and CMV infection, independent of other risk factors. The risk factors included in the logistic regression analysis were D+/R- CMV serostatus combination, alemtuzumab antirejection therapy, and relative duration of VGCV prophylaxis. The relative duration of prophylaxis was calculated as the duration of VGCV prophylaxis in days divided by the total number of days that VGCV should have lasted, based on the indication. These risk factors were prespecified based on biological plausibility. No univariate screening was performed to avoid overfitting the logistic regression model in accordance with statistical recommendations.^21^

To analyze leukocyte counts in the first post-transplant month, a linear mixed-effects model was fitted with random intercepts and slopes for individual patients. To evaluate differences between patients with and without DGF, DGF was included in the second model with a random intercept and slope. The correlation between log-transformed GCV exposure and leukocyte counts at different times after GCV measurement was assessed by calculating the Pearson correlation coefficient and visualized as a scatterplot with a linear regression line. Statistical significance is reported at *P* < 0.05. All statistical analyses were performed using R statistical software (v4.3.2).^22^

## RESULTS

### Patient Characteristics

A total of 353 patients were included, and 357 steady-state predose GCV concentrations were measured in these patients. Most measurements were obtained in the first and second weeks post-transplant (192 and 137 measurements, respectively). Most patients (94.9%) received induction therapy with basiliximab and received maintenance immunosuppression consisting of tacrolimus, mycophenolate mofetil, and prednisolone (92.9%). Twelve patients (3.4%) died within the first year after transplantation. Twenty-six cases of death-censored graft loss occurred during follow-up (7.4%). The median time to death-censored graft loss was 122 days (range, 7–287 days). The patient, donor, and transplantation baseline characteristics are available in the Supplemental Materials (see **Tables S1 and S2**, **Supplemental Digital Content 1**, http://links.lww.com/TDM/A875).

### CMV Infection

**FIGURE 1.**
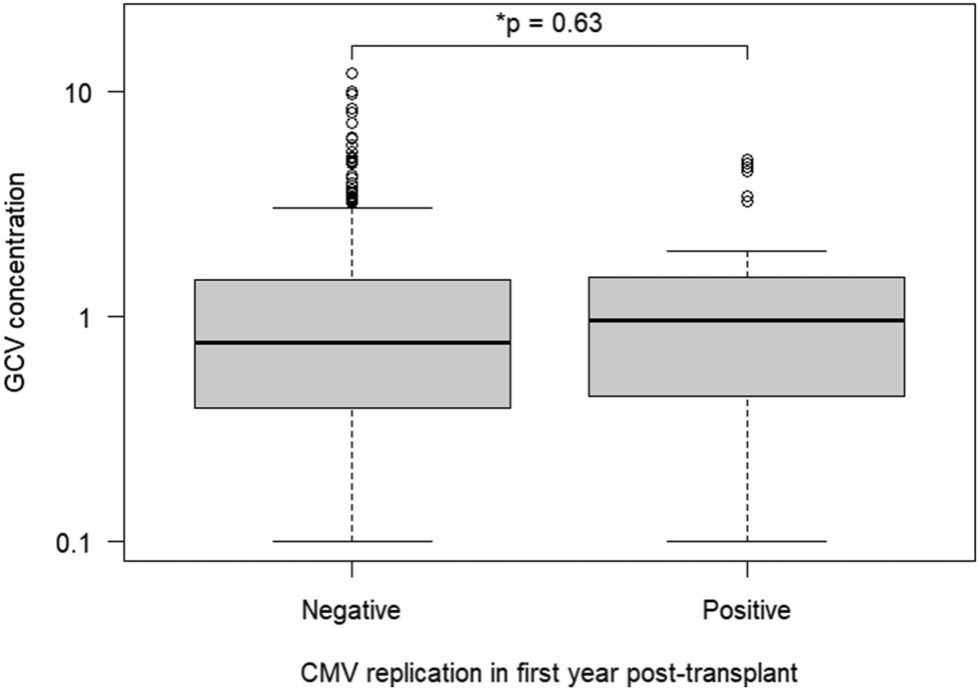
Early ganciclovir exposure in patients with and without CMV viremia during later follow-up. CMV, cytomegalovirus; GCV, ganciclovir.

CMV viremia was diagnosed in 30 patients (8.5%). Ten patients experienced a *primo* CMV infection, and 20 patients had CMV reactivation. Of these 30 patients, 13 had asymptomatic CMV viremia, and 17 (4.8%) had symptomatic CMV infection. Among the latter, only 2 patients suffered from organ-specific CMV disease: one patient had CMV colitis and one had CMV retinitis. The remaining 15 symptomatic patients were diagnosed with CMV syndrome. The median time from transplantation to CMV viremia was 207 days (range, 24–343 days). Eleven cases occurred during VGCV prophylaxis, compared with 19 cases that occurred after cessation of prophylaxis. Twenty-one patients were treated with therapeutic doses of VGCV; in 5 cases, only the immunosuppressive therapy was adapted, and in 4 cases, no specific treatment was initiated. The median duration of CMV viremia was 69 days (range, 7–299 days). GCV concentrations were not different between patients with CMV viremia (median 0.96 mg/L) and without viremia (median 0.76 mg/L, *P* = 0.63; Fig. 1).

### Ganciclovir Exposure and Kidney Function

**FIGURE 2.**
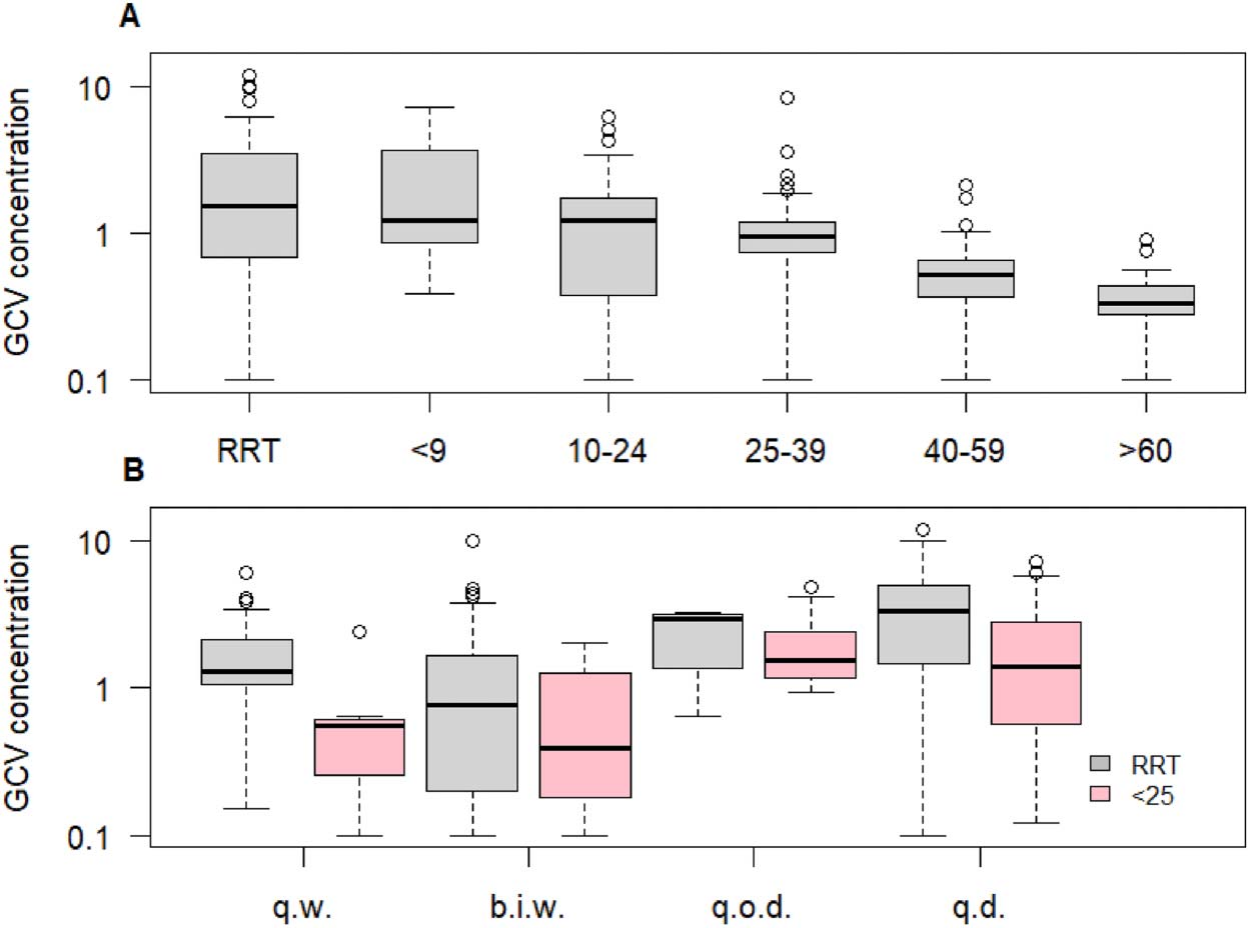
A, Variability of GCV exposure over different ranges of eGFR. B, Variability of GCV exposure for patients on renal replacement therapy and with kidney function below 25 mL/min/1.73 m^2^ according to the received VGCV dose. b.i.w., 450 mg twice per week; GCV, ganciclovir; q.d., 450 mg every day; q.o.d., 450 mg every other day; q.w., 450 mg once per week; RRT, renal replacement therapy.

Figure 2A shows that GCV exposure was the most variable for patients who were on renal replacement therapy (RRT) and those who had an eGFR <25 mL/min/1.73 m^2^. Figure 2B shows that this variability in GCV exposure was irrespective of the VGCV dose received by patients on RRT and with an eGFR below 25 mL/min/1.73 m^2^.

Table 1 summarizes the target attainment within the cohort, its relationship with eGFR, and the median daily dose of VGCV. Among the predose GCV concentrations, 236 were within target (66.1%), 106 were below target (29.7%), and 15 were above target (4.2%). Overall, there was a difference in target attainment among the different eGFR groups, with RRT as a separate category (Table 1, *P* < 0.001). *Post hoc* testing revealed a higher proportion of measurements above the target with RRT than without RRT (*P* < 0.001). Furthermore, there was a higher proportion of measurements below target with eGFR above 40 mL/min/1.73 m^2^, compared with below 40 mL/min/1.73 m^2^ and RRT (*P* < 0.001).

**TABLE 1.** Target Attainment of Predose Concentrations Per eGFR Group, With Median Daily Dose of VGCV

	Below Target (%) | Median Dose per day (mg)*		Within Target (%) | Median Dose per day (mg)*		Above Target (%) | Median Dose per day (mg)*	
RRT	19/92 (21)	129	63/92 (68)	129	10/92 (11)	450
<25 mL/min/1.73 m^2^	19/72 (26)	129	49/72 (68)	225	4/72 (5)	450
25–39 mL/min/1.73 m^2^	9/77 (12)	450	67/77 (87)	450	1/77 (1)	450
40–59 mL/min/1.73 m^2^	34/84 (40)	450	50/84 (60)	450	0 (0)	—
>60 mL/min/1.73 m^2^	25/32 (78)	450	7/32 (22)	450	0 (0)	—

* The median dose per day was calculated as the total dose over a specific dosing interval divided by the number of days of the dosing interval.

eGFR, estimated glomerular filtration rate; RRT, renal replacement therapy; VGCV, valganciclovir.

Overall, patients on RRT received relatively high median daily dosages of VGCV compared with the dosing guidelines of 100 mg 3 times per week (Table 1).^15^ Nevertheless, 21 percent of patients on RRT had GCV exposure below the target. The median daily dose for RRT patients with GCV exposure below target was not significantly different from that of patients with GCV exposure above target (*P* = 0.41). In contrast, the median daily dose in patients receiving RRT with GCV exposure above target was significantly higher than that in patients receiving GCV exposure at target (*P* = 0.016). For patients with an eGFR <25 mL/min/1.73 m^2^, the median daily dose to attain GCV exposure within the target range was also higher than recommended. There were no significant differences between the received median daily dose of patients with GCV exposure at target and patients who were below (*P* = 0.07) or above (*P* = 0.06) the target range. For all patients with an eGFR between 25 and 40 mL/min/1.73 m^2^, the median daily dose was 450 mg, irrespective of the target attainment category. This dose led to the smallest proportion of patients with below-target GCV exposure among all eGFR classes, and only 1% of patients within this eGFR class experienced exposure above the target range. For patients with an eGFR between 40 and 60 mL/min/1.73 m^2^, the median daily dose was 450 mg, which is in line with the current eGFR-based dosing guideline.^15^ Forty percent of these patients were underexposed to GCV. Patients with an eGFR above 60 mL/min/1.73 m^2^ were relatively underdosed (median daily dose of 450 mg instead of 900 mg, according to the eGFR-based dosing guideline),^15^ and 79% of these patients had a GCV exposure below target (Table 1).

### Delayed Graft Function

In total, 130 patients (36.8%) experienced DGF. The proportion of patients who developed CMV infection was not significantly different between those with DGF (9.2%) and those without DGF (8.1%, see **Table S3**, **Supplemental Digital Content 1**, http://links.lww.com/TDM/A875; *P* = 0.70). The occurrence of DGF was also not associated with CMV infection in multivariable analysis (OR 1.23, 95% confidence interval [CI] 0.55–2.68, *P* = 0.61; Table 2). Only D+/R-status was significantly associated with CMV infection (OR 3.07, 95% CI 1.7–7.06, *P* = 0.01; Table2).

**TABLE 2.** Multivariable Analysis of CMV Viremia

Variables	OR (95% CI)	*P*
Alemtuzumab antirejection therapy (no/yes)	1.62 (0.44–4.83)	0.42
DGF during first wk post-transplant (no/yes)	1.23 (0.55–2.68)	0.61
CMV D + R- status (no/yes)	3.07 (1.27–7.06)	0.010
Relative duration of CMV prophylaxis (in d)	1.01 (0.80–1.19)	0.90

CI, confidence interval; CMV, cytomegalovirus; D+, donor with positive CMV status; DGF, delayed graft function; R-, recipient with negative pretransplant CMV status.

**FIGURE 3.**
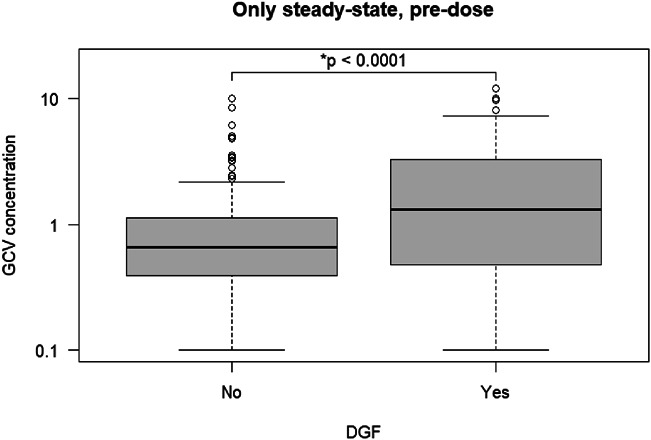
Early ganciclovir exposure in patients with and without delayed graft function after kidney transplantation. CMV, cytomegalovirus; DGF, delayed graft function.

Of the 357 steady-state predose concentrations, 116 samples were obtained from 116 patients during a DGF episode. Figure 3 shows that DGF was associated with higher GCV exposure (*P* < 0.001). The median GCV concentration during DGF was 1.32 mg/L, compared with 0.66 mg/L for samples obtained without DGF. There were also significant differences in target attainment between samples (see **Table S4**, **Supplemental Digital Content 1**, http://links.lww.com/TDM/A875, *P* = 0.003). *Post hoc* testing showed a higher proportion of measurements above the target during DGF (*P* = 0.001).

### Leukopenia

During the first month after transplantation, 37 (10.5%) patients developed leukopenia. Nineteen out of 130 (14.6%) patients with DGF developed leukopenia compared with 18 out of 223 (8.1%) without DGF, which was not significantly different (*P* = 0.07).

**Supplemental Digital Content 1** (see **Figure S1**, http://links.lww.com/TDM/A875) shows the mean leukocyte counts during the first post-transplant month for the entire population and for patients with and without DGF. GCV exposure was not associated with lower leukocyte counts on the day of GCV measurement (see **Figure S2A**, **Supplemental Digital Content 1**, http://links.lww.com/TDM/A875), or 1 day (see **Figure S2B**, **Supplemental Digital Content 1**, http://links.lww.com/TDM/A875), 3 days (see **Figure S2C**, **Supplemental Digital Content 1**, http://links.lww.com/TDM/A875), and 7 days (see **Figure S2D**, **Supplemental Digital Content 1**, http://links.lww.com/TDM/A875) after GCV measurement.

## DISCUSSION

Here, we present a real-world clinical experience of monitoring GCV exposure early after kidney transplantation. No association was observed between DGF or GCV exposure and CMV viremia. However, GCV exposure was highly variable early after kidney transplantation, especially in patients with estimated poor graft function and with nonfunctioning grafts. Overall, 25%–30% of patients were found to have GCV exposure below target, regardless of DGF, although many patients received higher doses than recommended for their estimated kidney function. Importantly, the incidence of CMV viremia and symptomatic disease was low at 8.5% and 4.8%, respectively, in the first post-transplant year, whereas only 10.5% of patients in the present cohort developed leukopenia during the first post-transplant month.

The association between DGF and CMV viremia reported by Kleinherenbrink et al was not confirmed in the current study. Their hypothesis that this association could be caused by GCV underexposure during DGF was also not substantiated by our findings, as patients with DGF were found to have higher GCV concentrations. In the present study, local practices may have led to higher dosing in patients experiencing DGF than that reported by Kleinherenbrink et al, which could have caused increased drug exposure within this group. We propose that this increased exposure may have contributed to the absence of an observed association between DGF and subsequent CMV viremia. However, as the current study evaluated only single GCV concentrations during DGF, this hypothesis remains speculative. Future research evaluating longitudinal exposure during DGF is necessary to test this theory.

The high variability in GCV exposure between individuals observed here can be explained by the variability in GCV pharmacokinetics, which was also reported in pharmacokinetic studies of GCV and VGCV. In these studies, despite adjusting the dose for the eGFR, variability was found both in the distribution volume and clearance of GCV.^23^ Furthermore, variable absorption has also been described after VGCV.^23^ We believe that another important factor that could have contributed to the high variability of GCV in this study is the difference between the eGFR and true kidney function in the dynamic situation shortly after kidney transplantation. Patients with eGFR in the same range could have had different true kidney functions, which would influence GCV clearance and thus GCV exposure. As this was a real-world study and no experiments were conducted, no actual measured GFRs were available to support this hypothesis. As the real-time measurement of true kidney function in dynamic situations is an invasive and costly procedure, it is unlikely that future studies will incorporate such measurements. Instead, individual monitoring of GCV exposure and clearance should be performed, as discussed below.

The clinical outcomes in the present cohort were good compared with those reported in other studies.^24^ The incidence of CMV viremia and symptomatic disease were both low compared with the incidence range of 5.2%–63.2% and 3.8%–19.0%, respectively, as reported by Silva Junior et al.^24^ This could indicate that the current dosing guidelines are adequate in the prevention of CMV despite frequent underexposure early after kidney transplantation. However, we cannot rule out the possibility that measuring the GCV exposure may lead to dose adjustments and better clinical outcomes. With respect to toxicity, incidence of leukopenia <3500 cells/μL was estimated 26% (95% CI 22–30) after 2 months with VGCV prophylaxis in adult kidney transplant recipients,^20^ which is higher than the extrapolated incidence of leukopenia in this cohort. The favorable clinical outcomes in the present cohort may be explained by higher GCV exposure, especially for patients with lower estimated kidney function, and by monitoring GCV exposure during the early dynamic phase after kidney transplantation, which may have led to timely dose adaptations in under- and overexposed patients. However, the low incidence of CMV viremia might also be explained by the discontinuation of prednisolone maintenance therapy after 5 months in most patients, the use of basiliximab as standard induction therapy, and the fact that patients were not routinely screened for CMV viremia. The local practice of tapering or temporarily discontinuing comedications such as mycophenolate mofetil and trimethoprim/sulfamethoxazole, which are also known to cause hematologic abnormalities after kidney transplantation,^25^ may also have contributed to the low leukopenia rates.

The strengths of the current study include the relatively large cohort size, the real-world setting, and the combination of relevant transplantation- and CMV-related outcomes with early GCV exposure. However, this study has some limitations. Most importantly, only singular GCV concentrations were available for each patient, and individual GCV exposure over time was unknown. Patients below or above the target may have been corrected for adequate exposure after this measurement; conversely, patients with adequate exposure could have experienced under- or overexposure at later time points, which remained unobserved. As both viral replication and the inhibition of leukocyte development take time, it is possible that only patients with repeated GCV exposure below or above target develop infection or leukopenia, respectively. In addition, no formal TDM protocol was used during the study period, and whether dose adjustments were made based on GCV measurements was not documented. Therefore, the effect of GCV measurements on the clinical outcomes could not be evaluated. Last, only GCV predose concentration was measured and not area-under-the-curve concentrations (AUC), which may correlate better with clinical outcomes.^4^

Future studies should focus on evaluating the longitudinal exposure to GCV, including AUCs, in the early post-transplant period and the effects of GCV concentration-based dose adjustments. If such studies confirm the high interindividual variability during this dynamic period and show that GCV exposure can be improved by concentration-guided dose adaptations, dosing guidelines for early after kidney transplantation should shift from eGFR-based dosing to GCV concentration-guided dose adaptations.

## CONCLUSIONS

Underexposure to GCV occurred frequently in the early period after kidney transplantation, with marked interindividual variability despite eGFR-based VGCV dosing. However, DGF was not associated with a reduced exposure to GCV or an increased risk of CMV viremia. Early GCV levels did not correlate with adverse CMV-related outcomes or leukopenia. These findings highlight the limitations of the current dosing strategies, the need for individualized dosing approaches, and the need to monitor GCV prophylaxis in kidney transplant patients.

## Supplementary Material

SUPPLEMENTARY MATERIAL
