# Supplementary material for: A Real-World Clinical Evaluation of Ganciclovir Exposure and Cytomegalovirus Viremia Outcomes After Adult Kidney Transplantation
Source: Ther Drug Monit. 2025 Jul 21;48(1):127–33. doi: 10.1097/FTD.0000000000001363 (PMC12771963; doi:10.1097/FTD.0000000000001363)
Supplement: SUPPLEMENTARY MATERIAL [file tdm-48-127-s001.docx]

**Table S1: Patient baseline characteristics**

| **Variables** | **Categories** | **n = 353** |
| --- | --- | --- |
| Recipient age in years | Median (IQR) | 62 (51 – 68) |
| Recipient sex | Female/Male (%) | 147/206 (42/58 |
| Recipient BMI at therapy | Median (IQR) | 26.8 (24.1 – 31.2) |
| Recipient CMV status | Negative/Positive (%) | 67/286 (19/81) |
| Primary underlying kidney disease | Diabetic nephropathy (%) | 62/353 (18) |
|  | Glomerulonephritis (%) | 65/353 (18) |
|  | Hypertensive nephropathy (%) | 81/353 (23) |
|  | Polycystic kidney disease (%) | 44/353 (12) |
|  | Reflux nephropathy/congenital (%) | 3/353 (1) |
|  | Unknown etiology (%) | 36/353 (10) |
|  | Other (%) | 62/353 (18) |
| Number of transplantations | 1/2/3 or more (%) | 295/40/18 (84/11/5) |
| Highest PRA (%) | Median (IQR) | 4 (4 – 5) |
| Pre-emptive transplantation | No/Yes | 256/97 (73/27) |
| HLA-A mismatch | 0/1/2 (%) | 101/174/76 (29/49/22) |
|  | Unknown | 2 |
| HLA-B mismatch | 0/1/2 (%) | 47/161/143 (13/46/41) |
|  | Unknown | 2 |
| HLA-DR mismatch | 0/1/2 (%) | 71/183/97 (20/52/27) |
|  | Unknown | 2 |
| BMI, body mass index; CMV, cytomegalovirus; HLA, human leukocyte antigen; IQR, interquartile range; PRA, panel-reactive antibodies. | | |
|  |  |  |

**Table S2: Donor and transplant characteristics**

| **Variables** | **Levels** | **n = 353** |
| --- | --- | --- |
| Donor age in years | Median (IQR) | 59 (49 – 68) |
| Donor sex | Female/Male (%) | 169/184 (48/52) |
| Expanded criteria donor | No/Yes (%) | 216/132 (62/38) |
|  | Unknown | 5 |
| Donor CMV status | Negative/Positive | 123/229 (34/65) |
|  | Unknown | 1 |
| CMV donor-recipient combination | D-R- | 8/352 (2) |
|  | D-R+ | 115/352 (33) |
|  | D+R- | 59/352 (17) |
|  | D+R+ | 170/352 (48) |
|  | Unknown | 1 |
| Donor type | DBD/DCD/Living | 73/155/125 (21/44/35) |
| Cold ischemia time (minutes) | Median (IQR) | 505 (136 – 733 ) |
| CMV, cytomegalovirus; D-, donor with negative CMV status; D+, donor with positive CMV status; DBD, donation after brain death; DCD, donation after circulatory death; IQR, interquartile range; R-, recipient with negative pre-transplant CMV status; R+, recipient with positive pre-transplant CMV status. | | |

**Table S3: CMV replication in patients with and without DGF**

|  | **DGF** | **No DGF** |
| --- | --- | --- |
| **CMV replication** | 12 (9.2%) | 18 (8.1%) |
| **No CMV replication** | 118 (90.8%) | 205 (91.9%) |

CMV, cytomegalovirus. DGF, delayed graft function.

**Table S4: Target attainment of pre-dose concentrations obtained during DGF *versus* not during DGF**

|  | **Below target (%)** | **Within target (%)** | **Above target (%)** |
| --- | --- | --- | --- |
| **During DGF** | 30 (25.9%) | 75 (64.7%) | 11 (9.5%) |
| **Not during DGF** | 76 (31.5%) | 161 (66.8%) | 4 (1.7%) |

DGF, delayed graft function.


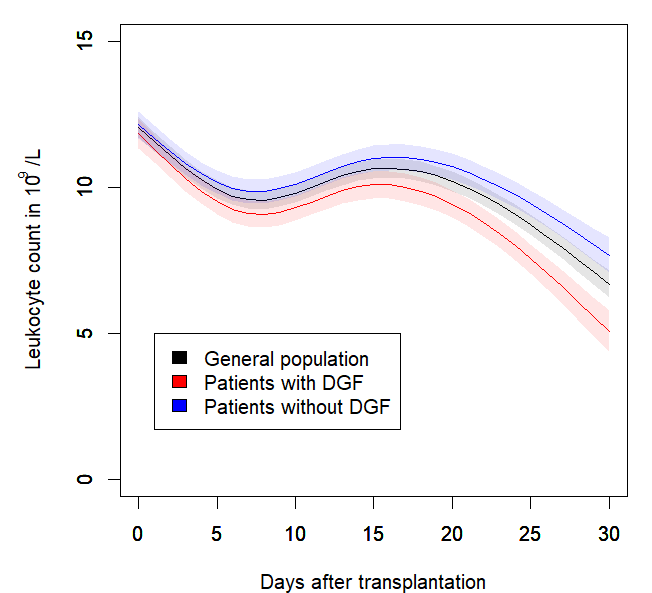


**Figure S1: Linear mixed effects model of leukocyte count over time in the general population and for patients with and without DGF.**

DGF, delayed graft function.

**
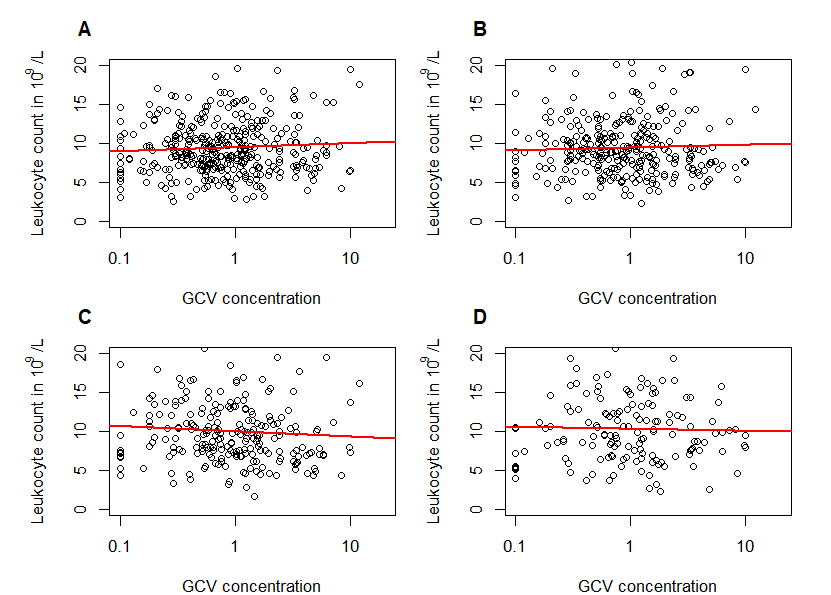
**

**Figure S2: Association between ganciclovir exposure and leukocyte counts at the day of ganciclovir measurement (A), 1 day (B), 3 days (C), and 7 days (D) after ganciclovir measurement.**
